# Personalising psychotherapies for depression using a novel mixed methods approach: an example from Morita therapy

**DOI:** 10.1186/s13063-019-3788-3

**Published:** 2020-01-08

**Authors:** Holly Victoria Rose Sugg, Julia Frost, David A. Richards

**Affiliations:** Institute of Health Research, University of Exeter Medical School, University of Exeter, Exeter, Devon UK

**Keywords:** Personalised treatment, Precision medicine, Mixed methods, Mental health, Psychotherapy, Morita therapy, Depression, Predictors

## Abstract

**Background:**

Current quantitative methods for personalising psychotherapies for depression are unlikely to be able to inform clinical decision-making for hundreds of years. Novel alternative methods to generate hypotheses for prospective testing are therefore required, and we showcase mixed methods as one such approach. By exploring patients’ perspectives in depth, and integrating qualitative and quantitative data at the level of the individual, we may identify new potential psychosocial predictors of psychotherapy outcomes, potentially informing the personalisation of depression treatment in a shorter timeframe. Using Morita therapy (a Japanese psychotherapy) as an exemplar, we thus explored how Morita therapy recipients’ views on treatment acceptability explain their adherence and response to treatment.

**Methods:**

The Morita trial incorporated a pilot randomised controlled trial of Morita therapy versus treatment as usual for depression, and post-treatment qualitative interviews. We recruited trial participants from general practice record searches in Devon, UK, and purposively sampled data from 16 participants for our mixed methods analysis. We developed typologies of participants’ views from our qualitative themes, and integrated these with quantitative data on number of sessions attended and whether participants responded to treatment in a joint typologies and statistics display. We enriched our analysis using participant vignettes to demonstrate each typology.

**Results:**

We demonstrated that (1) participants who could identify with the principles of Morita therapy typically responded to treatment, regardless of how many sessions they attended, whilst those whose orientation towards treatment was incompatible with Morita therapy did not respond to treatment, again regardless of treatment adherence and (2) participants whose personal circumstances impeded their opportunity to engage in Morita therapy attended the fewest sessions, though still benefitted from treatment if the principles resonated with them.

**Conclusions:**

We identified new potential relationships between “orientation” and outcomes, and “opportunity” and adherence, which could not have been identified using existing non-integrative methods. This mixed methods approach warrants replication in future trials and with other psychotherapies to generate hypotheses, based on typologies (or profiles) of patients for whom a treatment is more or less likely to be suitable, to be tested in prospective trials.

**Trial registration:**

Current Controlled Trials, ISRCTN17544090. Registered on 23 July 2015.

## Background

Depression is the most common mental health disorder and leading cause of disability worldwide [1–4]. Whilst evidence indicates that antidepressant medication (ADM) and several psychotherapies such as cognitive behavioural therapy (CBT) are, on average, equally effective in treating depression [5, 6], there is also much room for improvement: between one third and half of patients do not respond to treatment, and many do not adhere to treatment, thus impeding treatment effectiveness [7–15]. Indeed, modelling studies show that treatment can reduce the disease burden of depression in only approximately 33% of patients [16].

As individuals vary widely in response to specific treatments, one way to improve outcomes is to develop personalised depression treatments, or match patients to treatments, by identifying which individual patient characteristics predict treatment outcomes [17–19]. Personalised medicine is considered both a priority and a challenge for mental health researchers, and research on predictors and moderators of outcome is of vital importance [18–20].

Whilst research on differential response to ADM focuses on biomarkers, research on differential response to psychotherapies has largely focused on quantitatively measured clinical characteristics such as depression severity, history and subtypes; comorbid conditions and sociodemographic factors [17–19, 21]. However, such research has produced only limited knowledge about who benefits most from which treatment [17, 18], leaving only a “trial and error approach” towards depression treatment [22] (p.40). Efforts based on post hoc moderator analysis have been largely unsuccessful [21], and a recent review of randomised trials comparing two psychotherapies in patients with 27 specific characteristics indicates that completing sufficient trials to show an effect size of g = 0.50 would require another 326 years of research [23]. Understanding how combinations of such characteristics predict outcomes would require a longer timeframe still [19].

Authors therefore advocate alternative methodological approaches, especially those that can provide hypotheses to be tested in future trials [19, 23]. Whilst some progress has been made in developing predictive models combining various moderators to be tested in prospective trials (e.g. [24–26]), it can be argued therefore that what is needed is not more patient *numbers* but more *understandings*, to inform decision-making in a shorter timeframe*.*

Mixed methods may be one alternative methodological approach for generating such hypotheses. Rather than categorising patients according to quantified clinical and sociodemographic characteristics, using this approach we can be guided by the views of individual patients themselves: their attitudes, values and preferences in relation to treatment acceptability. This understanding of patients’ perspectives is key to personalising treatment; it can be argued that if we seek to individualise treatment, analysis should be at the level of the individual rather than the group [27] and augmenting quantitative approaches with a deeper dive into the rich, narrative data of individuals may enable us to best address individual complexity [28].

As such, we may obtain understandings of potential psychosocial predictors of treatment outcomes (or social biomarkers [29]) to inform the personalisation of psychotherapy for depression [30]. Whilst others argue for the importance of such factors, the role of patients’ views on treatment acceptability as a potential moderator of treatment outcomes has received little attention, and any such studies typically rely on quantitative measures alone [31–35]. However, qualitative and mixed methods have several potential advantages in this field. Qualitative methods are well-suited to the study of these social and experiential processes, which are imbued with personal meanings and difficult to express in quantitative terms [29, 36]. By taking an exploratory qualitative approach, unconstrained by predefined variables, and integrating qualitative and quantitative data at the level of the individual in a systematic and transparent manner [37], we may identify unexpected yet empirically derived variables based on patients’ perspectives, which potentially explain treatment outcomes, to be evaluated in future trials.

Using Morita therapy (MT) as an exemplar, we utilised this novel mixed methods approach in the Morita trial. The trial follows on from an iterative programme of work conducted to develop our MT clinical protocol, whereby we optimised MT according to the views of stakeholders [38]. MT [39] is a Japanese psychotherapy, informed by Zen Buddhist principles, with a holistic approach aiming to improve everyday functioning rather than targeting specific symptoms [40, 41]. Key components are outlined in Table 1. In the conceptual model underpinning MT, unpleasant thoughts and emotions are accepted as part of the natural ecology of the human experience, which ebb and flow as a matter of course and cannot be controlled by will. Accordingly, MT contrasts with the focus of established Western approaches, such as CBT, on symptom control [42].

**Table 1:** Key components of Morita therapy (MT)

	Components	Definition
Principles	Natural world	MT conceptualises unpleasant thoughts and emotions as part of natural human experience. It draws upon the natural world, and the place of humans within it, to emphasise that symptoms are not subject to the patient’s control, and will naturally ebb and flow with time
	Acceptance and allowance of internal states	All emotions and thoughts (internal states) are accepted as they are. Any attempts to control, resist, avoid or intervene in symptoms are considered to exacerbate them within a vicious cycle; therapists thus help patients to move away from symptom preoccupation and combat and towards acceptance and action-taking. Thus, the objectives are to shift attention and perspective, and move patients to a position of accepting and responding to phenomenological reality as it is, rather than controlling or “fixing” symptoms
	Normalisation	Therapists label internal states as “unpleasant” and “pleasant” but not “good” or “bad”. They emphasise that all emotions are natural, or normal, and will ebb and flow on their own so long as attempts are not made to resist them
	Fumon (inattention to symptoms)	Therapists, in an effort to shift patients’ attention away from symptom preoccupation and combat, will not focus on discussion or analysis of patients’ symptoms or their causes, but will “steer” the conversation towards action-taking and the external environment
Process/ practice	Diaries	Patients complete daily diaries on which therapists provide comments to facilitate an acceptance of internal states and refocus attention on action-taking and the external environment
	Four-phased model	Rest and action-taking are structured within 4 phases: (1) rest; (2) light repetitive activities; (3) more challenging activities; (4) social reintegration. The process is understood to aid experiential acceptance of the natural ebb and flow of internal states; re-orientate patients in nature; and refocus attention from the “self”/internal states to external reality
	Rest	MT seeks to potentiate patients’ natural healing capacities, in contrast to resisting and exacerbating symptoms. Patients sit with their internal states as they are, to learn how they naturally ebb and flow with time if left unattended, and to build a natural desire to take action
	Action-taking *with* symptoms	Patients learn to undertake purposeful and necessary action, with or without their symptoms; action which is driven by “desire for life” rather than a desire to change internal states. MT thus aims to improve everyday functioning in spite of symptoms, with symptoms reducing as a by-product of moving from a mood-oriented to purpose-oriented and action-based lifestyle

The Morita trial represented the first trial of MT in the UK: a feasibility study encompassing a pilot randomised controlled trial of MT plus treatment as usual (TAU) versus TAU alone, and embedded qualitative interviews. We established that a large-scale MT trial is feasible and that MT shows promise in treating depression. Our qualitative results highlighted that (1) the extent to which participants’ expectations and understandings of depression and its treatment (or their “orientation” towards treatment) were compatible with MT, thus enabling or hindering their identification with the principles of MT, was tied to the extent to which MT was perceived as acceptable and (2) there is a distinction between engaging with MT on this conceptual level and engaging with MT on an operational level, with some practical challenges identified. The protocol, quantitative and qualitative results are reported elsewhere [43–45].

In our mixed methods study, reported here, we developed typologies of participants based on their qualitative views regarding the acceptability of MT, and integrated these with quantitative data to explore why individual participants differed in terms of their adherence and response to MT. Our aim was to understand whether patients’ perspectives can help to explain treatment adherence and response, in order to continue our optimisation of MT, develop hypotheses to be tested in the process evaluation of a future trial, and, ultimately, inform the personalisation of treatment.

### Research questions

Our research questions are:

1. How do participants’ views about Morita therapy relate to the variability in the number of treatment sessions they attend?
2. How do participants’ views about Morita therapy relate to whether they respond to treatment (≥ 50% reduction in depressive symptoms (Patient Health Questionnaire 9 (PHQ-9) [46]) from baseline to follow up)?

## Methods

### Design and aim

We employed a mixed methods embedded design [47] guided by a pragmatic philosophy [37]. For the quantitative and qualitative components (reported elsewhere) [44, 45], we collected data concurrently and analysed data sequentially (with quantitative data informing our sampling of qualitative interviews for analysis). We gave these components equal priority and mixed them interactively at the design and analysis levels. Our aim was to explore how qualitative data on acceptability explains treatment adherence and response.

### Setting, recruitment and data collection

Our full quantitative and qualitative methods are reported separately [44, 45]; a summary is presented here to provide context for our mixed methods analysis. In the Morita trial, we recruited 68 participants with major depressive disorder, with or without anxiety disorder(s), through general practice record searches in Devon, UK and randomised them to receive TAU or TAU plus 8–12 sessions of MT delivered by trained therapists at the University of Exeter’s AccEPT clinic following our MT clinical protocol [38]. With the participant’s consent, we audio-recorded all therapy sessions.

We collected the following data at baseline and 4 months post-baseline: severity of depressive symptoms (PHQ-9) and generalised anxiety symptoms (Generalised Anxiety Disorder questionnaire 7 [48]); quality of life (Short Form 36 Health Survey Questionnaire [49] and Work and Social Adjustment Scale [50]) and attitudes (Morita Attitudinal Scale for Arugamama [51]). For MT participants, we recorded the number of therapy sessions attended and reason for ending treatment. We completed post-treatment semi-structured interviews with consenting MT participants (*n* = 28) to explore their views of MT using a topic guide based on recent mental health trials addressing similar questions [13, 52, 53], MT literature and our MT optimisation study findings [38]. With participants’ permission, interviews were audio-recorded and transcribed verbatim. We managed qualitative data in NVivo10 [54] and analysed data using framework analysis [55].

### Sampling

Using a nested sampling design, we analysed mixed methods data from a sub-sample of participants [56, 57]. Thus, we purposively selected “key informants” ([56] p.240) on acceptability according to the following theoretically driven criteria deemed important in answering our research questions [57, 58]: (1) treatment adherence and (2) treatment response. To achieve maximum variation according to these criteria [58], we intended to include a quota of three participants within each subgroup in the resulting sampling matrix (Table 2) [57]. Where a larger number of participants comprised a subgroup (i.e. those who completed and responded to treatment), we further purposively sampled participants to ensure representation across the following criteria: presence or not of generalised anxiety disorder at baseline; participants’ experience or not of CBT; participants’ gender and therapist. Through utilising a combination of probability and purposive sampling orientations within a strategy suited to mixed methods research, we thus aimed to both capture the breadth of views on acceptability and explore the depth and diversity of views within each subgroup [55, 59].

**Table 2:** Sample

		Adherence to treatment		
		Withdrew < 5 sessions	Withdrew ≥ 5 sessions	Completed treatment
Treatment response? (≥ 50% reduction in depressive symptoms (Patient Health Questionnaire-9) from baseline to follow-up)	Yes	*n* = 3	*n* = 1	*n* = 6
	No	*n* = 2	*n* = 3	*n* = 1

### Analysis

Following separate analyses of the quantitative and qualitative data (reported elsewhere) [44, 45], we developed typologies of participants’ views of MT from our qualitative themes. We developed these typologies along two continuums representing the acceptability of (1) the MT principles and (2) the MT process, reflecting the distinction between engaging with MT on conceptual and operational levels, which ran through our qualitative findings. For each typology, we developed example participant vignettes from the qualitative data to illustrate the key features that define that typology. We then integrated data in a joint typologies and statistics display [47]. In this display, organised by typology, we included quantitative data on the number of treatment sessions attended by each participant, the mean number of sessions attended by all participants within each typology, participants’ reasons for withdrawing from treatment and whether or not they demonstrated a response to treatment.

To guard against the possibility of alternative explanations for our findings, where the qualitative data suggested confusion related to particular components of therapy (such as participants’ understanding of the purpose of “rest”) we confirmed that therapists showed fidelity to the therapy protocol by reviewing audio-recordings of relevant therapy sessions.

We describe our study in line with mixed methods reporting guidelines (see Additional file 1 for completed GRAMMS checklist) [60].

## Results

We included data from 16 participants in our analysis (Table 2): all participants who did not complete and/or did not respond to treatment (*n* = 10) and 6 who completed and responded to treatment. Participant characteristics are provided in Table 3.

**Table 3:** Participant characteristics

Characteristic	Number (percemtage) unless otherwise stated Total *n* = 16
Gender	
Female	9 (56)
Age (years)	
Mean (SD)	48 (12)
Ethnic origin	
White British	16 (100)
Education	
No qualifications	1 (6)
GCSE or O Level	3 (19)
Post GCSE or O Level	4 (25)
Undergraduate degree	4 (25)
Postgraduate qualification or higher	4 (25)
Marital status	
Married or cohabiting	10 (63)
Number of children	
Mean (SD)	1 (1)
PHQ-9 (depression) score	
Mean (SD) at baseline	17 (5)
Mean (SD) at follow up	9 (7)
50% reduction in PHQ-9 score from baseline to follow up	10 (63)
Adherence to Morita therapy	
Number of sessions attended (mean (SD))	7 (4)
Completed treatment	7 (44)
Withdrew ≥ 5 sessions	4 (25)
Withdrew < 5 sessions	5 (31)
Morita therapist (of two available)	
Therapist 01	8 (50)
Secondary SCID diagnoses (at baseline)	
Any anxiety disorder	10 (63)
Generalised anxiety disorder	7 (44)
Panic disorder with agoraphobia	4 (25)
Panic disorder without agoraphobia	4 (25)
Social phobia	2 (13)
Post-traumatic stress disorder	1 (6)
Obsessive compulsive disorder	1 (6)
Previous psychotherapy/counselling (at least one course)	
Any psychotherapy (not including counselling)	12 (75)
Cognitive behavioural therapy	10 (63)
Mindfulness-based cognitive therapy	4 (25)
Eye movement desensitization and reprocessing	1 (6)
Other psychotherapy	4 (25)
Counselling	8 (50)

Percentages may not always total 100, due to rounding

*PHQ-9* Patient Health Questionnaire 9, *SCID* Structured Clinical Interview for DSM-IV disorders, *SD* standard deviation

**Fig. 1:**
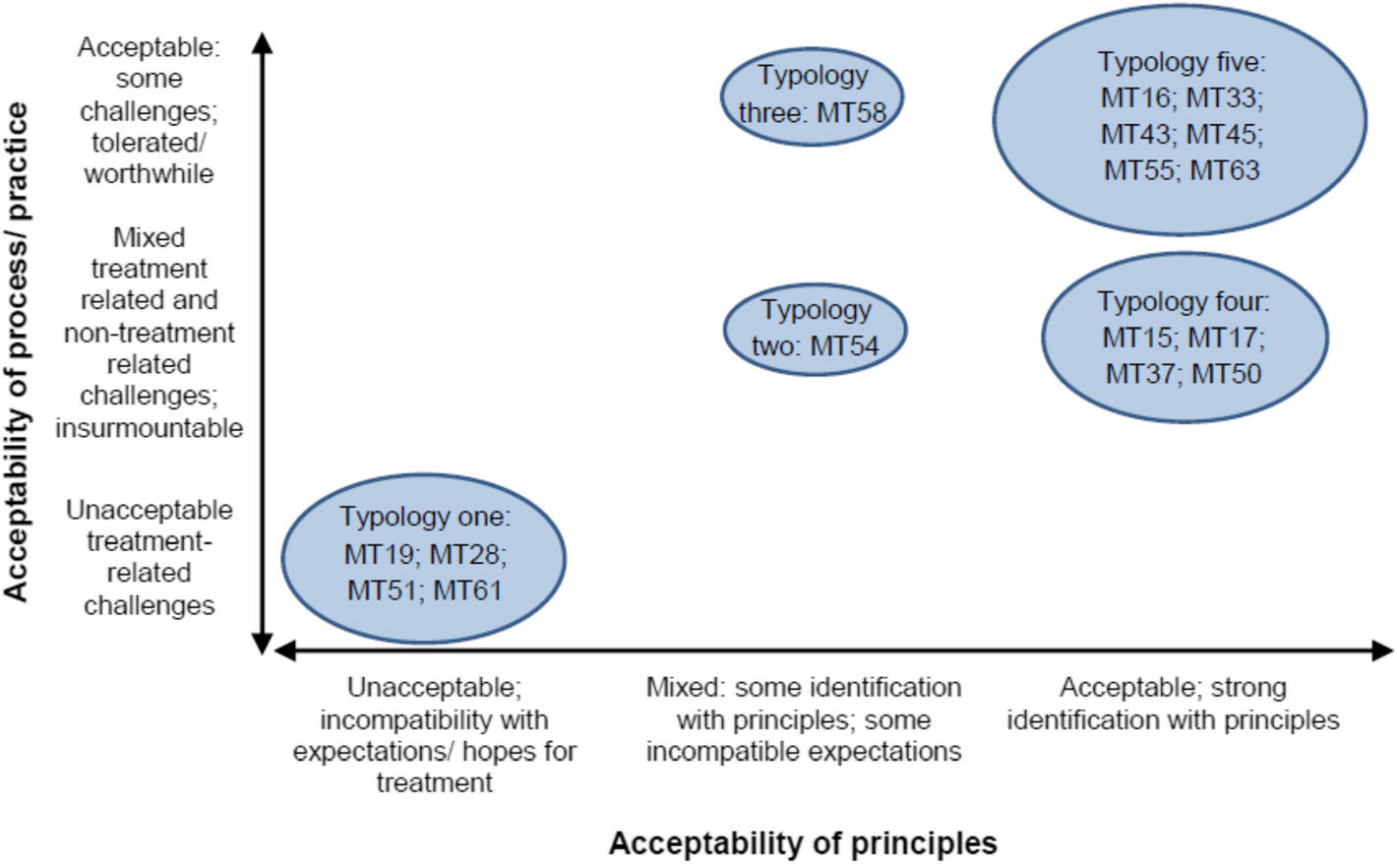
Typologies of acceptability

We developed five typologies (Fig. 1). In Fig. 1, the horizontal axis (from unacceptable principles to acceptable principles) represents the extent to which the MT principles (Table 1) were considered acceptable; the vertical axis (from unacceptable practice/process to acceptable practice/process) represents the extent to which the process and practice of MT (Table 1) were considered acceptable. The size of each typology represents the number of participants whose views fall within that typology. Following our joint display (Table 4), each typology is described in detail, in relation to the quantitative data and alongside example participant vignettes. Participants are referred to by trial ID number (MT__).

**Table 4:** Joint typologies (acceptability) and statistics (adherence) display

Typology of acceptability	Trial ID	Therapy sessions, number		Reason for withdrawing from treatment (N/A = completed treatment)	Treatment response?^a^
		Each	Mean		
(1) Principles: unacceptable (incompatibility with expectations/ hopes for treatment); process/practice: unacceptable (treatment-related challenges)	MT61	3	5	Discomfort with writing about self in diary; failure of rest to meet expected purpose	No
	MT19	5		Pressure of completing phases in absence of therapy fulfilling expected purpose	No
	MT51	5		Lack of techniques provided; challenges of rest in context of not fulfilling expected purpose	No
	MT28	7		Pressure of completing phases in absence of therapy fulfilling expected purpose	No
(2) Principles: mixed views (limited identification with principles); process/practice: mixed views (treatment related and non-treatment related challenges; insurmountable)	MT54	1	N/A	Time difficulties (rest/diary); difficulties with Fumon (therapists’ inattention to symptoms)	Yes (attributed to life changes)
(3) Principles: mixed views (limited identification with principles); process/practice: acceptable (some challenges; tolerated/worthwhile)	MT58	9	N/A	N/A	No
(4) Principles: acceptable (strong identification with principles); process/practice: mixed views (treatment related and non-treatment related challenges; insurmountable)	MT17	2	3.5	Time difficulties (rest)	No
	MT50	2		Safety issues (personal relationships) during rest	Yes
	MT15	3		Time difficulties (rest); no longer felt need for therapy	Yes
	MT37	7		Time difficulties (attending sessions); no longer felt need for therapy	Yes
(5) Principles: acceptable (strong identification with principles); process/practice: acceptable (some challenges; tolerated/ worthwhile)	MT33	9	10.8	N/A	Yes
	MT63	10		N/A	Yes
	MT45	11		N/A	Yes
	MT55	11		N/A	Yes
	MT16	12		N/A	Yes
	MT43	12		N/A	Yes

*N/A* not applicable

^a^Treatment response defined as ≥ 50% reduction in depressive symptoms (Patient Health Questionnaire-9 (PHQ-9)) from baseline to follow up

### Typology 1: principles unacceptable; process/practice unacceptable

The typology that appears at the bottom left of Fig. 1 represents the views of participants (MT19; MT28; MT51; MT61) who considered both the principles and practice of MT unacceptable. These participants all expressed an orientation towards treatment (expectations or understandings of depression and its treatment) that was incompatible with MT, such as seeking a cure for symptoms or in-depth self-analysis. They also expressed challenges of engaging with MT, which they considered insurmountable, such as the pressure of completing activities associated with the treatment phases. Rarely were such challenges expressed as insurmountable *because* of participants’ demanding personal circumstances, such as a lack of time; rather, these participants focused on the challenges of treatment regardless of their circumstances and often in the context of the treatment components failing to achieve the purpose assigned to them in relation to the participant’s particular orientation towards treatment (such as helping them to control symptoms).

Example vignette. MT61 approached treatment seeking an opportunity to “open-up”, and answers to enable them to stop unpleasant thoughts and feelings. MT61 struggled to identify with the MT principles: neither the ebb and flow of emotions nor understanding emotions through reference to nature resonated for them. MT61 misunderstood the purpose of rest as an opportunity for the therapist to analyse their sleep, potentially to understand more about them on an unconscious level, and considered it unrealistic to schedule and report on their sleep in this way. MT61 discontinued treatment after three sessions due to the discomfort of writing about themselves in the diary, in the context of disliking themselves.

These participants who found both the principles and practice unacceptable attended, on average, 5 treatment sessions (range 3–7) of a maximum of 12 before discontinuing treatment (for treatment-related reasons) (Table 4). None responded to treatment.

### Typology 5: principles acceptable; process/practice acceptable

In contrast to typology 1, the typology that appears at the top right of Fig. 1 represents the views of participants (MT16; MT33; MT43; MT45; MT55; MT63) who considered both the principles and practice of MT acceptable. These participants all identified with and were receptive to the MT principles, finding they resonated with their experiences and views of depression. In addition, whilst typically expressing some challenges of engaging in treatment such as the discomfort of “sitting with” unpleasant emotions during rest, these participants considered them tolerable and worthwhile. These views appeared to be facilitated by accurate understandings of the purpose of these treatment components as part of a progressive process for learning and re-focusing attention.

Example vignette. MT63 identified strongly with the underlying premise of understanding unpleasant thoughts and emotions as part of the natural human experience. Whilst noting that sitting with their thoughts was “terrifying”, MT63 understood the purpose of rest and learned the futility of engaging in the vicious cycle, as per their normal coping strategies, therefore considering these challenges worthwhile. MT63 also described the diary and spending time in nature in terms of learning how all things naturally pass. MT63 appreciated MT as a gentle, natural process of self-discovery, noting the value of an experiential approach, which had visceral, emotional and intellectual impact. MT63 experienced benefits of treatment in terms of normalising difficulties; increasing action-taking; decreasing self-criticism and symptoms. Compared with other treatments, MT63 felt that MT had fundamentally changed their attitude towards and acceptance of difficulties, as opposed to providing strategies for tackling symptoms which potentially “feed into” the vicious cycle.

These participants who found both the principles and practice acceptable attended, on average, 10.8 treatment sessions (range 9–12) (Table 4). All completed and responded to treatment.

### Typology 4: principles acceptable; mixed views on process/practice

The typology that appears on the right of the x axis and middle of the y axis of Fig. 1 represents the views of participants (MT15; MT17; MT37; MT50) who, whilst similarly identifying with the MT principles as per typology 5, experienced more significant challenges with the MT process. Typically, these challenges related to the time commitment and the discomfort associated with rest. These participants considered these challenges insurmountable *in the context of* their personal circumstances, such as other commitments or a lack of safety and social support during rest. Thus, unlike typology 1, these participants were keen to continue MT (which connected to their identification with the principles) and accurately understood the treatment components as part of a process, but found it unfeasible to engage with MT at this point in their lives.

Example vignette. MT50 found that the principles strongly resonated with them: understanding human emotion as cyclic and through reference to nature, both learning to be with unpleasant internal states and learning to take small steps of action, and nurturing one’s authentic self. Accordingly, MT50 hoped that treatment would help them to be more at ease with their feelings and to take more action in line with their values. MT50 expressed an accurate understanding of the purpose of therapy as a process for learning. MT50 engaged in one day of rest and, whilst describing some discomfort, noted a valuable lesson learned in terms of realising unpleasant internal states come and go. MT50 wanted to continue therapy but withdrew after two sessions as they felt unsafe resting at home in the context of a threatening neighbour. MT50 expressed significant benefits of treatment in terms of knowing symptoms will pass (like the weather), thus having more acceptance and less fear of them, consequently finding they pass more quickly. MT50 considered MT a “philosophy for life” and other treatments as short-term fixes that risk highlighting and exacerbating symptoms.

These participants who found the principles acceptable and practice unacceptable withdrew because of the practical challenges of engaging with MT in the context of their personal circumstances, alongside at times feeling that they no longer required therapy (Table 4). They attended, on average, 3.5 treatment sessions (range 2–7) and all but one (MT17) responded to treatment, although MT17 did show an improvement in symptoms.

### Typologies 2 and 3: mixed views on principles

Within the sample of interviews analysed, the typologies in the middle of the x axis of Fig. 1 were more anomalous, representing two participants (MT58; MT54) who expressed some identification with and/or positive views of the principles alongside some expectations and hopes for treatment that were incompatible with MT.

Firstly, MT54 (typology 2: mixed views on process/practice) expressed interest in the ideas of MT and in therapy involving a process. However, MT54 had been receiving private counselling which, if affordable, they would have continued. As such, MT54 struggled with Fumon (therapists’ inattention to symptoms), feeling that this “shut them down” and inhibited their rapport with the therapist. MT54 also expressed significant challenges associated with the time commitment of rest and diary completion in the context of childcare commitments. MT54 discontinued treatment for these reasons after attending one session (Table 4). MT54 responded to treatment but attributed this to changed life circumstances rather than treatment.

Secondly, MT58 (typology 3: process/practice acceptable) expressed identification with certain principles such as the vicious cycle. However, MT58 also sought to overcome their difficulties and tended to isolate each treatment component as a potential tool for tackling or distracting from symptoms. MT58 expressed challenges of engaging with MT related to these inaccurate understandings of the purpose, such as struggling to “shut out” thoughts during rest. However, MT58 considered the challenges tolerable and did not refer to difficulties in the context of demanding personal circumstances. MT58 indicated some benefits of treatment, such as reduced engagement in the vicious cycle, but intended to seek counselling and hoped that they would overcome their difficulties in time. MT58 completed treatment at nine sessions but did not respond to treatment (Table 4).

## Discussion

We found that our novel mixed methods approach can identify potential predictors of treatment outcomes, based on an individual’s attitudes and circumstances, which could not be derived from existing non-integrative methods for personalising depression treatment. In our example, participants who could identify with the MT principles typically responded to treatment regardless of the number of sessions they attended; conversely, those whose orientation towards treatment was incompatible with MT did not respond to treatment, again regardless of treatment adherence. Participants whose personal circumstances impeded their opportunity to engage in treatment generally attended the fewest number of sessions.

Thus, participants who considered both the MT principles and process unacceptable (associated with holding expectations or understandings of depression and its treatment that are incompatible with MT) discontinued treatment at between one and seven sessions; none responded to treatment. Participants who considered both the MT principles and process acceptable (expressing a strong identification with the principles alongside some worthwhile practical challenges) attended the most sessions; all responded to treatment. Participants with mixed views on acceptability (expressing a strong identification with the principles yet significant challenges of engaging with the process given their personal circumstances) discontinued treatment having attended the fewest sessions; however, they typically responded to treatment.

### Strengths and limitations

A key strength of this study is that it we have integrated our quantitative and qualitative data at the level of the individual and at the point of analysis, as opposed to at the point of discussion (as per typical mixed methods studies) [37]. Our identification of potential relationships between acceptability, adherence and response would not have been possible from a separate examination of the group-based quantitative and qualitative results alone, and is unlikely to have been possible from a comparison of such results within only the discussion. Furthermore, by integrating individual-level quantitative and qualitative data at the point of analysis in a systematic, transparent and rigorous manner, we produce conclusions that can be readily traced, understood and interrogated [37]. We further enriched our analysis and the meaningfulness of our findings by including participant vignettes and a confirmation of therapist fidelity where relevant, and we describe our study in line with mixed methods reporting guidelines [60].

In light of the current dearth of explicit examples of both the use and usefulness of integrative mixed methods analysis [61, 62], we therefore provide an example of how research questions can be designed to specifically address the integration of data, how such integration can be undertaken with rigour, and how such integration can generate additional learning, thus adding value through producing “a whole … that is greater than the sum of the individual qualitative and quantitative parts” [61] (p.116).

A potential limitation of this study relates to the number and range of cases included. Whilst little guidance is currently available on the appropriate sample size for mixed methods analysis, it is likely that considerations of the study purpose and heterogeneity of data are relevant. In this study, the overall sample size, and the sample size of each subgroup (e.g. those who completed but did not respond to treatment), was constrained by the number of participants in the Morita trial who fulfilled our sampling criteria. However, we purposively and explicitly selected participants in order to achieve maximum variation along the target dimensions of our mixed methods analysis (i.e. treatment adherence and response), with only additional participants who completed and responded to treatment not sampled. Nonetheless, our results are based on a limited amount of data and may not reflect the relationship between acceptability and outcomes in full, or be transferable to other contexts such as different psychotherapies.

### Clinical implications

Whilst our findings regarding orientation and opportunity as potential predictors of outcomes in MT will inform our process evaluation within any large-scale trial of MT [63], they also provide us with tentative insights that warrant further investigation in relation to other psychotherapies for depression. Consistent with our findings regarding participants’ orientation towards treatment, other studies (whilst not including mixed methods analysis) have suggested the importance of matching patient perceptions and expectations to the conceptual model underlying psychotherapy (including CBT, psychodynamic therapy and behavioural activation) [31, 64]. Whilst our findings suggest that patients who identify with allowing (as opposed to controlling) internal states may be more likely to respond to MT, others suggest the opposite pattern may be present in cognitive therapy [65], which would be consistent with the contrasting principles of these approaches. Thus, whether the degree of concordance between a patient’s orientation and the conceptual model of the treatment approach may predict treatment response, and potentially guide the matching of individuals to different psychotherapies, warrants further investigation.

Furthermore, noteworthy within our current findings is that this concordance appeared to override treatment adherence in explaining treatment outcomes: participants who identified with the principles of MT typically responded to treatment regardless of the number of sessions they attended (and vice versa). Though highly speculative, this suggests the potential importance of patients *engaging* with (the premise of) psychotherapy over and above *adhering* to psychotherapy by rote. Distinguishing between engagement and adherence in psychotherapy in terms of their relationship to outcomes is an area for further research.

If replicated, our findings might also inform the tailoring of specific psychotherapies, in terms of the optimal treatment “dose” for different patients. In MT, we might tailor the approach to the needs of patients who identify with the principles but experience demanding personal circumstances (thus impeding their opportunity to continue MT) by developing a form of “low-intensity” MT, comparable to low-intensity CBT [66], with reduced engagement in the four treatment phases. Thus, we may ultimately develop a clinical algorithm whereby patients are matched to MT on the basis of their compatible orientation towards treatment, and stratified to low-intensity or high-intensity MT on the basis of their personal circumstances. More widely, whilst the current movement towards reducing therapy intensity is driven primarily by cost-savings to improve overall access to therapy [67], our findings point to the potential for a more coherent rationale based on the views and needs of patients themselves. Thus, the provision of low-intensity options for suitable patients may constitute “minimally disruptive medicine”: a necessary and beneficial reduction in the burden of treatment based on the realities of (certain) patients’ lives [68] (p.1).

### Methodological implications

The development of personalised treatments is a major priority for mental health researchers, yet current methods are unlikely to be able to inform clinical decision-making for hundreds of years [20, 23]. We have showcased mixed methods research as an alternative and novel methodological approach, which includes a deep exploration of patients’ perspectives and the integration of qualitative and quantitative data at the level of the individual. By focusing on these individual-level data and not being constrained by predefined variables, this exploratory method can enable us to identify new and unexpected potential predictors of treatment outcomes to be tested in prospective trials; predictors that are empirically-driven (unlike many of those currently examined in trials [23]) and that could not be identified using existing non-integrative methods. Our mixed methods approach can therefore meet a current need in the personalisation of treatment: the development of hypotheses for future testing, in order to personalise depression treatment in a shorter timeframe [23].

Furthermore, through focusing on patients’ views, values and circumstances in relation to treatment acceptability, mixed methods can identify potential *psychosocial* predictors of treatment outcomes, combined in a meaningful way in the form of typologies (or profiles) of patients for whom a treatment may be more or less suitable. Such psychosocial factors have received relatively little attention in personalising treatment, despite potentially playing an important role in improving our currently limited understanding of not only *whether* a patient with a certain characteristic will or will not adhere/respond to treatment, but *why* [29, 34, 69]. We therefore recommend that mixed methods analysis be incorporated into trials of other psychotherapies for depression, in order to generate hypotheses for testing in prospective trials, and ultimately inform both the matching of different treatments to patients and the tailoring of specific treatments to patients on the basis of the attitudes and circumstances of individual patients themselves.

## Conclusions

Our novel mixed methods approach can inform personalised trials and treatments by identifying potential predictors of treatment outcomes that would be missed by existing non-integrative approaches. With current quantitative methods unlikely to be able to inform clinical decision-making for hundreds of years, we argue for this consideration of depth rather than breadth in the personalisation of treatment: integrating rich qualitative data (requiring smaller patient numbers) with quantitative data at the level of the individual in order to identify unexpected potential psychosocial predictors of treatment outcomes is an alternative worth pursuing. We therefore recommend the replication of these methods with other psychotherapies for depression, to investigate whether key potential predictors in Morita therapy (participants’ orientation and opportunity) may apply across treatments, and with a view to continued hypothesis generation for testing in prospective trials. Thus, this methodological approach may inform the development of both better trials and better treatments, and, ultimately, the personalisation of psychotherapies based on the attitudes and circumstances of individual patients.

## Supplementary information

**Additional file 1.** Good Reporting of A Mixed Methods Study (GRAMMS) checklist. Completed GRAMMS checklist with page numbers of included items.

## Data Availability

The datasets used and/or analysed during the current study are available from the corresponding author on reasonable request.

## Abbreviations

ADM: Antidepressant medication
CBT: Cognitive behavioural therapy
MT: Morita therapy
PHQ-9: Patient Health Questionnaire 9
TAU: Treatment as usual
