# Supplementary material for: Personalising psychotherapies for depression using a novel mixed methods approach: an example from Morita therapy
Source: Trials. 2020 Jan 8;21:41. doi: 10.1186/s13063-019-3788-3 (PMC6950935; doi:10.1186/s13063-019-3788-3)
Supplement: Supplementary file 1 — Additional file 1. Good Reporting of A Mixed Methods Study (GRAMMS) checklist. Completed GRAMMS checklist with page numbers of included items. [file 13063_2019_3788_MOESM1_ESM.docx]

**Additional file 1. Good Reporting of A Mixed Methods Study (GRAMMS) checklist.**

| **Guideline** | **Section: page** |
| --- | --- |
| Describe the justification for using a mixed methods approach to the research question | Introduction: p.5-6 |
| Describe the design in terms of the purpose, priority and sequence of methods | Design: p.9 |
| Describe each method in terms of sampling, data collection and analysis | Setting, recruitment and data collection: p.9-10 |
| Describe where integration has occurred, how it has occurred and who has participated in it | Sampling: p.10; Analysis: p.11 |
| Describe any limitation of one method associated with the present of the other method | Strengths and limitations: p.21 |
| Describe any insights gained from mixing or integrating methods | Discussion: p.19-24 |

Template from O'Cathain A, Murphy E, Nicholl J. The quality of mixed methods studies in health services research. J Health Serv Res Policy. 2008;13(2):92-98.
